# Cardiac troponin and defining myocardial infarction

**DOI:** 10.1093/cvr/cvaa331

**Published:** 2021-01-17

**Authors:** Thomas E Kaier, Bashir Alaour, Michael Marber

**Affiliations:** King’s College London BHF Centre, The Rayne Institute, 4th Floor, Lambeth Wing, St Thomas’ Hospital, Westminster Bridge Road, London SE1 7EH, UK

**Keywords:** Troponin, Myocardial infarction, Myocardial injury

## Abstract

The 4th Universal Definition of Myocardial Infarction has stimulated considerable debate since its publication in 2018. The intention was to define the types of myocardial injury through the lens of their underpinning pathophysiology. In this review, we discuss how the 4th Universal Definition of Myocardial Infarction defines infarction and injury and the necessary pragmatic adjustments that appear in clinical guidelines to maximize triage of real-world patients.

## 1. Introduction

The measurement of Cardiac troponin concentration in systemic venous blood has become a core component of the assessment of patients with acute—and chronic—cardiovascular disease. This is enshrined in the Universal Definition of Myocardial Infarction (UDMI),^1^^,^^2^ now in its fourth iteration—with the aim to (i) guide the clinician through the numerous differential diagnoses that result in cardiac troponin elevation, and (ii) provide classification and naming conventions to assist a structured approach. However, the 4th UDMI has stimulated considerable debate.^3–5^ Our previous review^6^ covered the basic biology of cardiac troponin, the physiology underlying its release from the heart, the analytic science enabling its detection in the blood, and its use in the diagnosis of myocardial infarction according to the 3rd UDMI.^7^ The purpose of this current review is to discuss the pathophysiology that underpins the 4th UDMI and how it is translated into clinical guidelines and practice—with a specific focus on the challenges encountered ‘at the coalface’ of acute cardiovascular care.

## 2. Summary of the 4th UDMI

The 4th UDMI is based on sound pathophysiological concepts which are then used to classify everyday cardiovascular events in patients with or without diagnostic ST-elevation on their presenting ECG. Such patients are almost always first identified by a troponin concentration in a venous blood draw exceeding the ‘normal’ range; defined by the 99th centile upper reference limit (URL). For reasons that are practical, rather than rational, patients in whom myocardial infarction is extremely unlikely will still have their troponin measured. Although, such overuse of the troponin assays seems benign, from a Bayesian perspective it means that the pre-test probability of myocardial infarction is very low. Depending on the specific healthcare environment studied, this results in wide variation in prevalence of myocardial infarction—from 5% to 20% in published studies across the world.^8^^,^^9^

**Figure 1:**
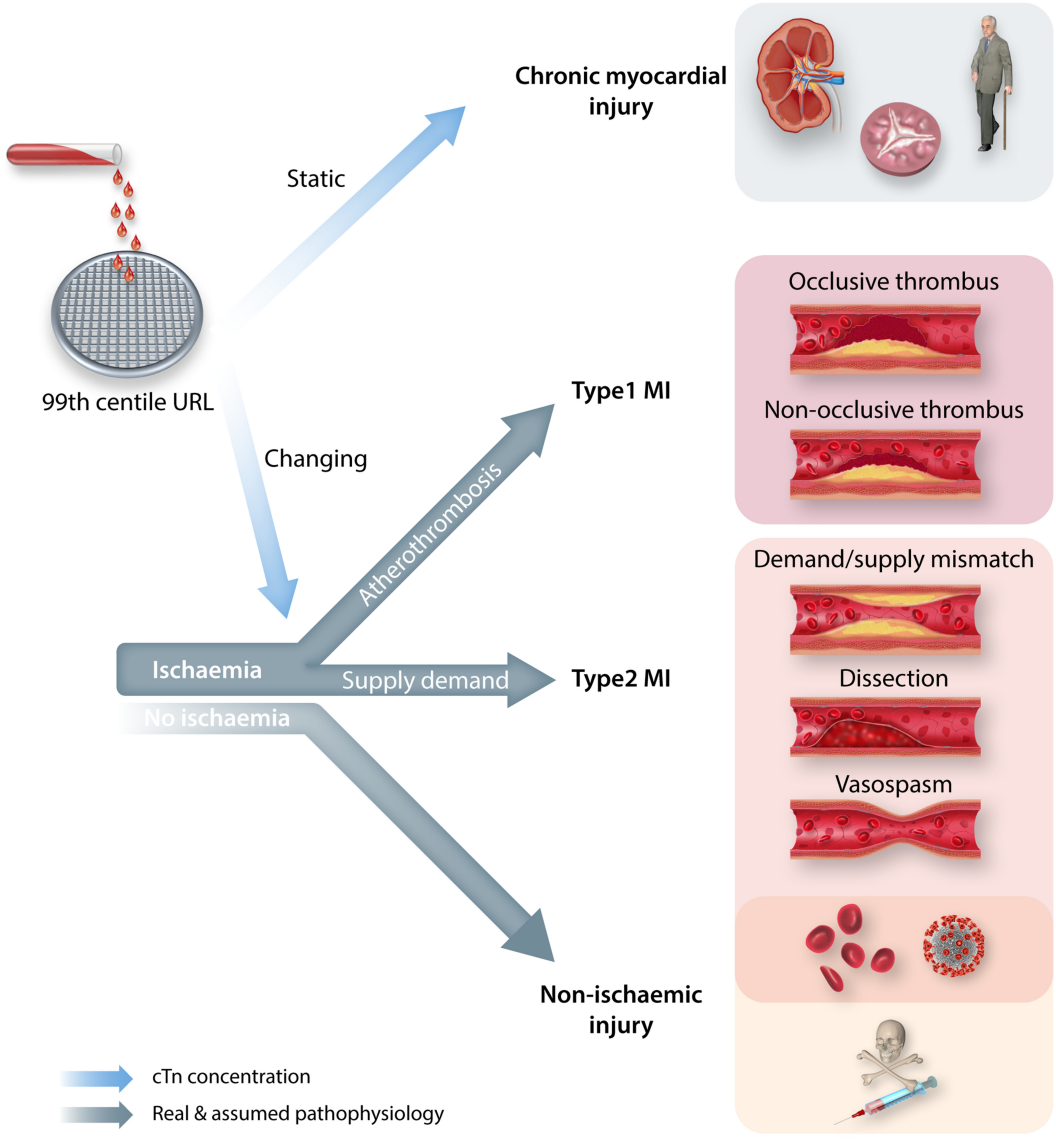
Schematic of the 4th Universal Definition of Myocardial Infarction (UDMI). The blood sample is from a patient with suspected non-ST elevation acute coronary syndrome. The sieve represents the cardiac troponin concentration cut-point at the 99th centile upper reference limit. In patients with troponin above this concentration, the UDMI recommends a differentiation of myocardial infarction from injury, and emphasizes the use of the best imaging techniques available to define aetiology of myocardial injury (preferably cardiac magnetic resonance imaging).^1^ See text for further details.

The 4th UDMI therefore follows a ‘surgical sieve’ approach in an attempt to filter these heterogeneous patient cohorts towards their correct classification. This sieve applies three questions in series (see *Figure 1*): (i) Is the concentration of cTn above or below the 99th centile URL? (ii) Is the cTn concentration static or changing? (iii) Is there evidence of myocardial ischaemia?

All patients with a cardiac Troponin concentration above the URL are defined as having ongoing myocardial damage—in those with a dynamic rise/fall the damage is considered to be acute/unstable—in those with more static concentrations the damage is considered to be chronic/stable. Those patients with acute damage are defined as having either acute myocardial infarction (AMI) or acute myocardial (non-ischaemic) injury, with the dichotomy between these conditions driven by the presence or absence of cardiac ischaemia, respectively. The following are considered indications of cardiac ischaemia:

- signs (ECG) or symptoms of myocardial ischaemia,
- new loss of viable myocardium,
- evidence of coronary thrombus, and
- the context of the cTn elevation (spontaneous, peri-procedural).

The 4th UDMI then subclassifies AMI based on aetiology—with Type 1 MI referring to events triggered by atherosclerotic plaque rupture/erosion. Type 2 MI can be seen as a hypernym capturing all the events and processes that lead to myocardial ischaemia not caused by acute atherosclerotic plaque rupture. These include excessive myocardial demand and/or reductions in myocardial supply (such as coronary artery vasospasm, microvascular dysfunction, coronary embolus, or spontaneous coronary artery dissection) in the presence or absence of stable obstructive atherosclerosis. The other subclassifications of AMI are more straightforward—Type 3 MI occurs in patients who suffer cardiac death due to likely myocardial ischaemia. Type 4a MI is myocardial infarction occurring in the context of percutaneous coronary intervention (PCI), Type 4b MI is due to stent/scaffold thrombosis following prior PCI, Type 4c MI is due to restenosis (within stent or in the native coronary artery following plain old balloon angioplasty); finally Type 5 MI occurs in the setting of coronary artery bypass graft (CABG) surgery. For types 4 and 5 AMI, a cTn threshold much higher than the 99th URL is used to reduce the incidence of trivial AMI and just highlight events that maybe clinically meaningful. The use of a higher cTn concentration for procedural vs. spontaneous AMI is controversial and has recently been reviewed elsewhere (see E.H.J. Bulluck *et al*.,^2^ in review).

Although this framework to filter patients towards their final diagnosis is logical and straightforward, its translation into clinical practice is confounded by the reality of cardiovascular pathophysiology. Below we discuss these key confounders in the order that they arise in *Figure 1*.

### 2.1 What has changed from the 3rd UDMI?

The 4th UDMI has introduced novel concepts: Most importantly, there is now a clear distinction between myocardial infarction and injury, with the latter also affecting the classification of peri-procedural injury. New sections include the description of Takotsubo syndrome and Myocardial Infarction with Non-Obstructive Coronary Arteries (MINOCA). The document further touches on the use of non-invasive imaging techniques to assist in defining aetiology of injury (CMR) and anatomy in the context of infarction (CT coronary angiography). Several concepts have seen an update, as described above; most importantly, sex-specific 99th centile thresholds (which are assay-specific) received a clear recommendation where hs-cTn assays are used.

## 3. Overview of troponin structure and risk of false-positives

The structure of the sarcomere as the contractile unit of the heart has been discussed previously (see Chapters 2.1 and 2.2 of Park *et al*.^6^). In brief, the different subunits of cardiac troponin^10^, specifically troponin T and I which are targeted by the monoclonal antibodies used in modern cTn assays, were first described and classified by Greaser and Gergely in 1973:^11^ the *inhibitory* fraction (cTnI) limits activity of the actomyosin ATPase, while the cTnT fraction binds to *tropomyosin* and serves as a mechanical link. Modern hs-cTn assays specifically quantify cardiac isoforms of the troponin subunits, but there is evidence to suggest that re-expression of foetal isoforms can occur in the context of pathologies affecting the skeletal muscle.^12^ These have been shown to cross-react with the (cardiac) monoclonal antibodies, thus yielding a true false-positive result in a rare set of conditions.^13^ Assay interferences from endogenous sources comprise (rare) endogenous antibodies against either the cTn complex or the exogenous, usually murine, antibodies used for detection and/or capture cTn.^14^ Biotin in over-the-counter dietary supplements can interfere with biotin–streptavidin based assays: depending on the assay format (competitive vs. sandwich method), this can result in false-negative or false-positive results.^15^

## 4. Release mechanisms

Experimental evidence supports the thesis that release of cTn is exclusively due to irreversible cell death.^16–18^ It was postulated that myocardial ischaemia alone could result in cTn release without evidence of necrosis—potentially with only ‘reversible’ cell damage or through the release of membranous blebs; neither mechanism is substantiated to date.^19–21^ For an in-depth review of these mechanisms, we refer the reader to Chapter 6.1 of Park *et al*.^6^ Overall, there appears to be a spectrum of ischaemia/reperfusion injury—some mild enough to remain sub-clinical—but it appears unlikely that there is *no* myocyte death when there is cTn detection.^22^ After all, the latest hs-cTn assays operate in the femtomolar range, thus providing greater resolution and precision than modern imaging techniques.^23^^,^^24^ Furthermore, depending on the timing and success of revascularization following myocardial infarction, evidence suggests extended elevation of cTn for 12–20 days.^25^

## 5. The 99th centile URL

The 99th centile is the threshold to distinguish between ‘normal’ and ‘abnormal’ cTn concentrations. The dichotomy is artificial since the mode, median, mean, range, and skewness of the distribution of troponin concentrations in a given ‘healthy normal’ population depends on the inclusion criteria used to define ‘normal’. As Apple *et al*.^16^ point out, these criteria vary between assay manufacturers—whereas some define *healthy* based on age (<30 years) and apparent lack of comorbidity, others screen with blood tests (e.g. for natriuretic peptides to exclude sub-clinical cardiac dysfunction) and group according to race and sex. These inclusion criteria are not standardized and they each ‘shift’ the 99th centile, which increases with age, male sex, serum creatinine (renal dysfunction), and a range of other cardiovascular risk factors.^26^ One ramification of ill-defined 99th centiles was highlighted by Shah *et al*., amongst others, who compared a combined 99th centile to sex-specific thresholds [which are almost two-fold higher in men than in women for hs-cTnI (Architect)], demonstrating that the combined threshold would result in an under-diagnosis of AMI in women.^16^^,^^27–29^ But the use of sex-specific thresholds for the 99th centile is not without debate—and has demonstrated significant variation depending on the cTn assay in use. In a systematic review published by Kimenai *et al*.,^30^ hs-cTnI was subject to much higher variation (80%) with respect to the 99th centile thresholds than hs-cTnT (29.4%). Nevertheless, the female-specific URL was consistently lower than the uniform decision limits for any hs-cTn assay. In a large cluster-randomized controlled trial involving >48 000 patients, hs-cTnI with sex-specific threshold identified 5 times more women than men with any myocardial injury, however without an improvement in outcomes; the latter possibly due to undertreatment.^31^ Interestingly, in a retrospective analysis of hs-cTnT data collected as part of a prospective diagnostic multicenter study, the use of sex-specific thresholds did not lead to a significant diagnostic reclassification.^32^ Overall, the variation appears to affect hs-cTnI assays more than hs-cTnT, with a risk/benefit assessment favouring the use of sex-specific thresholds—likely a contributing factor to the 4th UDMI’s endorsement of their use.^1^

With respect to standardization, Apple *et al*.^33^ summarized recommendations for defining an assay-specific 99th centile on behalf of the International Federation of Clinical Chemistry and Laboratory Medicine (IFCC) Task Force on Clinical Applications of Cardiac Bio-Markers (TF-CB). Hopefully, these recommendations will increase uniformity of the 99th centile URL across assay platforms. In addition to population selection, the assay method and specimen type can also influence the 99th centile. Thus, the minimum recommended sample size for derivation is ‘300 male and 300 female subjects’.^33^ As per the IFCC recommendations, a hs-cTn assay ought to (i) achieve an imprecision defined by the coefficient of variation (CV) ≤10% at the 99th centile and (ii) quantify cTn in ≥50% of healthy subjects. Both requirements encourage the use of less stringent inclusion criteria for normal studies; which will skew the concentration vs. frequency distribution to the right and increase the 99th centile URL.

Crucially, the population of patients undergoing troponin testing *on clinical grounds* includes many individuals who would have been excluded from the ‘healthy’ normal population used to derive the 99th centile URL.^34^ As a direct consequence, the prevalence of cTn concentrations above the URL increases from the expected 1% to as much as 40%. This very high ‘false-positive’ prevalence of myocardial injury is a major challenge for the clinical implementation of high-sensitivity assays and the way in which this liability is mitigated is discussed further below.

## 6. Static or changing troponin?

The cTn concentration measured in a healthy stable person varies overtime due to a combination of measurement imprecision (analytical variance) and true biological variation. cTnI concentrations appear to vary randomly over a 24-h period: the coefficient of variation within-subject (CV_I_) is constant at 8–9% for all time intervals and is unaffected by the underlying renal function.^35^^,^^36^ cTnT on the other hand follows a marked diurnal variation in healthy volunteers, with the concentration in the morning on an average 4 ng/L (approximately 1/3rd of URL) higher than in the evening.^36^

As discussed, much more than 1% of the population presenting to emergency departments will have a cTn concentration above the 99th centile URL even when they were stable, outside hospital, and going about their daily activities. This is because they are older, have more cardiovascular risk factors, and worse renal function than the healthy reference population used to define the 99th centile URL. It is therefore necessary to distinguish people with chronic/stable elevations in cTn concentration from those with acute elevations related to a medical event triggering their presentation. Those with chronically elevated troponins may have absolute cTn concentrations which are similar to those in patients with acute, but minor, myocardial injury; so, magnitude alone cannot be used to differentiate between these scenarios. As the concentration of cTn increases the chances of an underlying acute event rise too. Nonetheless, cTn concentration alone is poor at discriminating acute from chronic myocardial damage, particularly with concentrations of cTn modestly above the 99th centile URL. The two groups can also be distinguished by identifying a change in concentration over time that exceeds the ‘noise’ expected in stable individuals due to a combination of natural biological variation and the analytic variance of the assay. The need to identify that the cTn concentration is either rising and/or falling is an absolute requirement for the diagnosis of myocardial infarction that is endorsed in all the major guidelines.^37^^,^^38^ The most usual way to set the dichotomy limit between static or changing cTn is to compare concentration measurements from two blood draws separated by a defined time interval in patients in whom the final diagnosis has been rigorously classified. The change in the second troponin concentration relative to the first can either be expressed as an absolute difference or as a percentage. Reichlin *et al*.^39^ compared these different reference change strategies in a well-classified cohort and found absolute change to be diagnostically superior.

The guideline-recommended reference (or delta) change values are unexpectedly small [e.g. 3 ng/L for hs-cTnT (Elecsys), 2 ng/L for hs-cTnI (Architect)], however, these are optimized/calibrated to achieve a high sensitivity: As outlined, small fluctuations in cTn concentration occur over a 24-h period as well as in a diurnal pattern,^35^^,^^36^ and a number of assays demonstrate a substantial within-subject coefficient of variation when comparing short-term repeats.^40–42^ Consequently, diurnal and/or individual variation can result in false-positives for acute myocardial injury that compound the false-positives for any form of myocardial injury set by a 99th centile URL derived from a healthy cohort.

**Figure 2:**
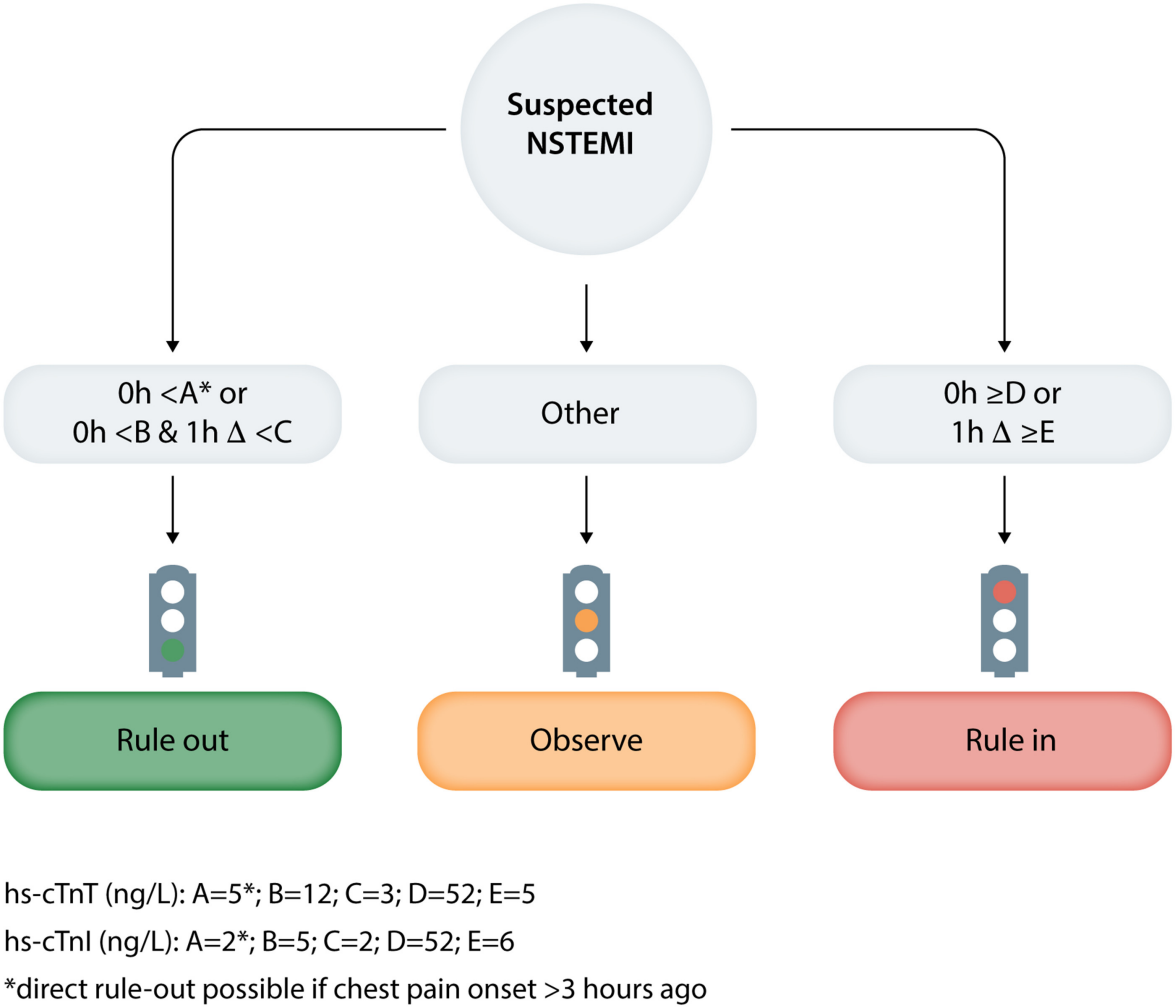
ESC 0/1 h rule-in and rule-out algorithms using high-sensitivity cardiac troponins (hs-cTn) assays in patients presenting with suspected non-ST-elevation myocardial infarction (NSTEMI) to the emergency department; concept as per Roffi *et al*.^38^

How do these considerations impact medical care? For this, we reference *Figure 2* (2015 ESC guidelines for the management of ACS^38^) which outlines how clinicians in the Emergency Department may assess patients for an acute coronary syndrome, in the absence of ST segment elevation on the ECG. As is evident from the flowchart, the decision cTn concentrations for immediate rule-out or rule-in of ACS on a single blood draw taken at presentation (0 h)—at least 3 h after chest pain onset for rule-out—are widely spaced around the 99th centile URLs of the commercial assays [14 ng/L for hs-cTnT (Elecsys), 34 ng/L in men and 16 ng/L in women for hs-cTnI (Architect)]. For those patients with cTn concentrations between these widely spaced limits a further blood draw is necessary after 1 h (other side of the ‘or’ in *Figure 2*). The absolute deltas in cTn concentration between first and second blood-draw, that are used to categorize patients are relatively low, only very minimal changes will allow the rule-out of an ACS. A greater change (≥5 ng/L for hs-cTnT, ≥6 ng/L for hs-cTnI) will categorize the patient as rule-in for ACS and likely lead to treatment for Type 1 MI with antiplatelet and anti-thrombotic medication and probable invasive coronary angiography with a view to PCI (balloon angioplasty and stent). In addition, evident from the flowchart is the intermediate (amber) group of patients that neither rule-in nor rule-out—who are confined to an ‘observe’ category. This accounts for the inherent uncertainty that occurs between the extremes of the immediate and 0/1-h decision thresholds and reflects the spectrum of human pathobiology. In fact, the decision thresholds are optimized for high sensitivity at the rule-out threshold, and high specificity at the rule-in threshold. As a consequence, 24–50% of patients assessed with the ESC 0/1 h algorithm remain in the ‘observe’ zone after a second blood draw^9^^,^^43–63^ and require further assessment.

### 6.1 Imaging adjuncts to refine risk stratification in the grey zones of hs-cTn pathways

The form of the assessment for those patients left in the observe zone has not yet been similarly protocolized and tends to be tailored to the individual patients. However, patients in the observe zone tend to be categorized further based on repeat cTn measurement, serial ECGs, and non-invasive imaging including CT coronary angiography (CTCA) and stress echocardiography. Only few studies to date have investigated the use of ECG-gated CTCA to refine the population with indeterminate hs-cTn concentrations—a majority of the evidence base stems from the era of sensitive cardiac troponin assays and is thus not translatable to modern chest pain protocols. Of those employing CTCA in conjunction with hs-cTn, the BEACON trial demonstrated no reduction in length of stay nor 30-day revascularization;^64^ Smulders *et al*.^65^ demonstrated a reduction of the use of invasive coronary angiography with similar outcomes when used in patients with hs-cTn concentrations above the 99th centile; the VERDICT trial evaluated the use of CTCA in an observational component of a trial comparing very early to standard invasive coronary angiography, demonstrating comparable diagnostic accuracy to rule-out significant coronary artery disease.^66^ Most recently, the 2020 ESC guidelines for the management of acute coronary syndromes recommend the use of CT as an alternative to invasive angiography ‘when there is low-to-intermediate likelihood of CAD’ and biomarkers are ‘normal or inconclusive’.^67^

Importantly, many of the patients in observe zone, as well as some patients in the Rule-In zone (up to 25%), will receive a final diagnosis of Type 2 MI or ‘chronic myocardial injury’. Latest data, adjudicated on the 4th UDMI, demonstrates that both are prognostically relevant and meaningful. But whilst there is compelling evidence that myocardial injury has a prognostic impact on individuals, in the presence or the absence of an acute event, the management of patients with chronic elevations is poorly defined and it is often unclear how their risk can be modified.^68–72^ We hence discuss the aetiology of chronic myocardial injury in greater detail below.

## 7. Chronic myocardial injury

The release of cTn is agnostic to the cause of myocardial damage. Why the majority of the healthy population has quantifiable cTn in their peripheral blood (based on high-sensitivity assays) remains an enigma. With the IFCC’s definition of high-sensitivity cardiac Troponin, it follows that a majority of individuals investigated at Emergency Departments have a cTn result above the LOD, and many above the URL.^33^^,^^73^ As such, the distinction of acute vs. chronic myocardial injury lies in both the magnitude of the cTn concentration and its temporality of change—this is illustrated by the derivation and validation of the cTn concentration cut points that underpin *Figure 2*. Below we summarize the chronic pathophysiological processes that are known to elevate cTn and may help shed light on why chronic cTn concentration correlates with long-term prognosis, even below the 99th centile URL.^74^^,^^75^

In the absence of a preceding acute insult, apoptosis can lead to chronic cTn elevation, in particular in the context of a failing heart.^76^^,^^77^ Hibernating myocardium might play a role in the chronic elevation of cTn but is both poorly understood and unproven. Inflammatory cytokines or increased plasma membrane permeability of injured cells might also play a role.^78^ Myocardial cell stretch-related mechanisms in viable and non-injured cardiomyocytes mediated by integrin signalling could further lead to constant cTn release.^79^

In the recovery period following an acute insult, remodelling of the injured myocardium ensues over weeks to months leading to a variable degree of tissue repair, compensatory hypertrophy, and replacement-fibrosis—which could explain cTn release that persists following MI.^80^ Left ventricular wall strain, interstitial changes reducing capillary density and resulting subendocardial ischaemia (due to lower coronary reserve), as well as activation of vasoconstrictive neurohormones could all contribute to cTn release under this circumstance.^81–86^

An interesting subset is the patient with stable coronary artery disease—recently coined ‘chronic coronary syndrome’ by the ESC^87^ as a juxtaposition to the acute syndrome causing infarction. At this stage, it is likely that a combination of different mechanisms leads to chronically elevated cTn concentrations in this cohort:^86^^,^^88–90^ apoptosis, cardiomyocyte turnover, myocardial strain, increased cardiac mass, and subclinical plaque rupture are all thought to contribute. The extent of coronary atherosclerosis and high-risk plaque phenotypes (based on intravascular ultrasound) also associate with elevated circulating cTn concentrations.^91^

Response to normal everyday physiological stress further adds to the pathological potpourri contributing to chronic myocardial injury. Numerous studies have detected elevated cTn concentrations in otherwise healthy individuals following physical exertion.^92–100^ Intriguingly, the magnitude of biomarker change poorly correlates with conventional cardiac risk factors. However, younger age,^101^^,^^102^ increased intensity of exercise,^97^^,^^99^^,^^102^ and lower baseline fitness^92^^,^^101^ are all significantly associated with the magnitude of cTn elevation post-exercise. Whilst there is no evidence of structural myocardial damage on cardiac MRI,^95^^,^^103–105^ this technique routinely lacks the resolution to identify the small volume myocardial damage that can cause significant cTn elevation—although, future applications using 7T and imaging optimization might be able to overcome these shortfalls.^24^^,^^106^ It was hypothesized that the cTn release during exercise could result from the release of cytosolic pools of cTn due to increased cellular permeability without irreversible myocardial injury.^93^^,^^98^^,^^100^ However, this concept is difficult to prove since cTn is the most sensitive marker of myocardial injury at our disposal and there is no alternative technique to arbitrate the presence or absence of reversibility.

## 8. The quest for ischaemia

The distinction between acute and chronic myocardial injury, based on whether cTn concentration is static or changing, is logical; albeit more complicated in clinical practice than predicted by pathological theory. The next key question to the right of the sieve in *Figure 1* is whether a cTn concentration above the 99th centile which is changing is due to myocardial injury or myocardial infarction? The latter mandates the presence of myocardial ischaemia at some point during the patient’s presenting illness. Unlike, the questions of whether cTn concentrations are changing or static, the dichotomy between acute myocardial injury and infarction is ephemeral.

Clinicians rely on relatively crude tools to detect the presence of ischaemia in the acute setting—chiefly, the pattern of chest pain symptoms and/or the occurrence of ST-segment changes on the surface ECG. The differential diagnosis of chest pain is very wide and includes common pathologies unrelated to the heart (e.g. musculoskeletal and oesophageal). Whilst the ECG—in the absence of ST elevation—has a sensitivity for AMI of less than 50%^107^ and poor specificity. Tools for the detection of stress-induced ischaemia are available, but these are designed and validated to quantify ischaemia in patients with stable coronary artery disease^87^ and cannot be used to detect ischaemia after a spontaneous event.

The gold-standard in the assessment of the coronary arterial tree—invasive coronary angiography—detects luminal narrowing, but cannot reliably identify or exclude an atherosclerotic plaque as the culprit of an acute coronary syndrome.^108^^,^^109^ The best intravascular imaging modalities use either intravascular ultrasound (IVUS) or optical coherence tomography (OCT). The former, in its conventional form, can reliably quantify lumen area, plaque burden, and vascular remodelling, but due to its axial resolution (70–200 µm) it cannot define plaque morphology.^110^ Near-infrared spectroscopy-IVUS (NIRS-IVUS) enhances conventional IVUS through its ability to further characterize a plaque’s lipid core.^111^ OCT provides higher resolution (10 µm) and has been used extensively for plaque characterization.^112–114^ Serial intravascular imaging studies have confirmed the development of atherosclerotic plaque occurs over several years, and morphological features such as cap thinning, plaque burden^115^ and microcalcifications together with shear stress—rather than luminal narrowing—determine the chance of plaque rupture.^109^ Therein lies the challenge of detection, as these vulnerable plaques often remain clinically silent due to their non-obstructive nature, until sudden plaque rupture and subsequent localized thrombosis leads to an acute coronary syndrome.

## 9. Evidence is required—of plaque rupture and atherothrombosis

In ST-segment elevation myocardial infarction, it is common to find thrombus (at least partially) occluding an epicardial coronary vessel on invasive coronary angiography.^116^ In only very few additional cases, the ‘evidence’ is as obvious—the ischaemic insult can trigger ventricular fibrillation, which leads to cardiac arrest and—unless promptly resuscitated—death.^117^ In patients with non-ST-elevation myocardial infarction (NSTEMI), and as outlined above, the ‘trail of evidence’ is much less clear. Most individuals will have suffered prolonged (>20 min) ischaemic chest pain at rest, but reveal a normal cardiovascular examination and unhelpful ECG.^38^^,^^118^ Beyond patient demographics and the assessment of cardiac biomarkers, there is little that can refine the clinician’s pre-test probability before proceeding to an invasive assessment of the coronary anatomy. The invasive coronary angiogram (ICA) can demonstrate localized (non-occlusive) thrombus, but more often demonstrates only luminal narrowing. Features suggestive of acute plaque rupture are intraluminal filling defects (consistent with thrombus), plaque ulceration (contrast holdup and hazy contour extending beyond the vessel lumen), irregularity of the plaque, dissection, or impaired flow.^38^^,^^119–121^ As many of these features are subject to intra-observer variability, identifying the culprit lesion remains a frequently encountered challenge in NSTEMI presentations. Intravascular imaging can improve the precision, but adds time, cost, and risk to the procedure which has to be carefully weighed against the potential benefits.

## 10. Demand and supply imbalance, chronic overload, and cellular injury

**Figure 3:**
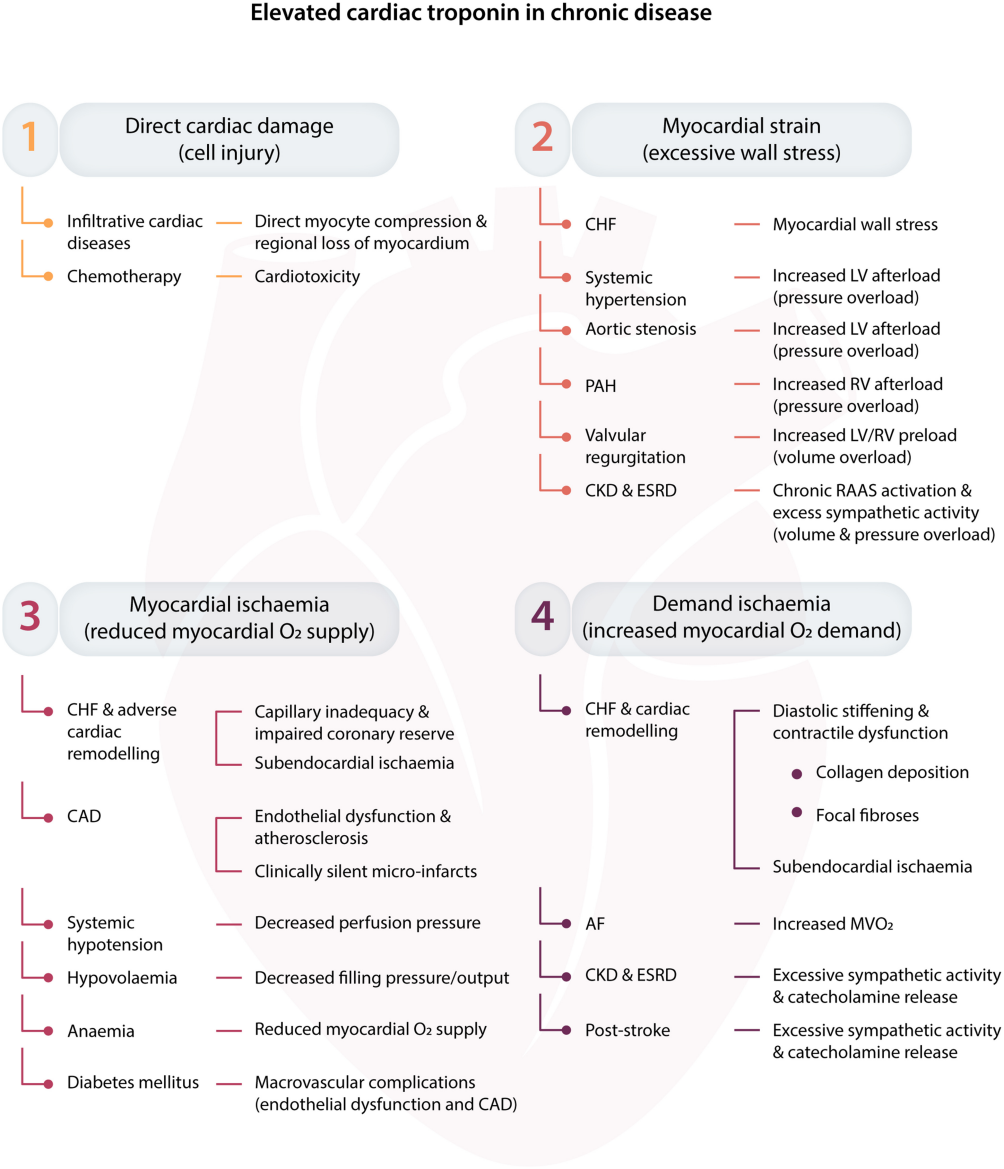
An outline of the different mechanisms contributing to an elevated cardiac Troponin concentration in chronic disease; from Park *et al*.^6^

Any transient insult to the myocardium will result in an acute biomarker release—depending on the circumstance, this might be an acute on chronic release when there are other factors contributing to myocardial strain. *Figure 3* highlights the many patterns of increased demand, ischaemia, strain, or direct cardiac damage which can lead to cardiac Troponin release.^6^ From a pathophysiological standpoint, there is significant overlap between conditions that cause chronic and acute cTn release and indeed those mechanisms have to be recognized as a continuous spectrum rather than an easily dichotomized disease entity.

## 11. Acute myocardial injury

There is one final disease classification that captures conditions with a dynamic cTn rise/fall above the 99th centile: acute myocardial injury. This might arise as a consequence of conditions such as myocarditis or non-cardiac entities such as renal failure—the implication being that there is no evidence of ischaemia leading to cellular damage and in some cases necrosis. The suggested mechanisms mirror those causing chronic myocardial injury, but due to the ‘acuity’ of the extra-cardiac insult, the heart is affected too—through mechanical stretch, physiological stress, apoptosis, increased turnover of cardiomyocytes, or cellular release of cTn degradation products.^1^^,^^122–124^ Given the challenges with reliable detection of ischaemia, the hypothesized margins between acute cTn release due to acute heart failure (acute myocardial injury) and severe hypertension (T2MI) certainly blur—as both would cause an increase in myocardial strain and affect myocardial wall stress, the pathophysiological differences are all but clear.

## 12. Why are clinicians unhappy?

The 4th UDMI takes an approach centred around the aetiology of cardiac Troponin elevation to classify disease entities. The challenge lies in the real-world application of these criteria—which require a *post hoc* analysis of all available clinical information to distinguish between categories such as Type 1 and Type 2 MI. And here is where the water becomes increasingly muddied—how does the clinician distinguish between a plaque-rupture event causing non-occlusive thrombus leading to distal embolization and subsequent cardiomyocyte necrosis^125^^,^^126^—Type 1 MI; and an oxygen supply/demand imbalance due to atherosclerosis—Type 2 MI—after the fact? Only a few milligrams of tissue are required to undergo necrosis to elevate the systemic cardiac Troponin level above the 99th centile,^24^ but the best available imaging modalities—cardiac magnetic resonance imaging—does not have the spatial resolution to discern supply/demand mismatch from distal embolization affecting less than 1 g of myocardium.^127^

Further complications arise from the mixed entities summarized as leading to Type 2 MI. Whilst non-atherosclerotic coronary dissection is classified as Type 2 MI, the process by which this causes myocardial necrosis is partially comparable to atherosclerotic plaque rupture resulting in obstructive thrombus formation (Type 1 MI): the lack of distal coronary blood flow for a transient period of time leads to necrosis, and the clinical management until the point of diagnosis remains similar—consideration of antiplatelet medication to limit an assumed pro-thrombotic state, and invasive coronary angiography to establish the exact cause of the clinical and biochemical syndromes.

The various pathologies which can be summarized under Type 2 MI yield an additional challenge: whilst an update of the International Classification of Diseases (10th edition) has introduced a code for Type 2 MI,^128^ the heterogeneity of the classification makes future research incredibly challenging. De Lemos *et al*.^3^ have highlighted that at least three entities (SCAD, coronary embolism, and vasospasm) are acute processes that require management similar to Type 1 MI. As such, their recommendation was to re-classify the above entities under a sub-group of Type 1 MI—to allow a distinction between atherosclerotic events and acute coronary obstruction for other reasons. Whilst treatment for these diagnoses are different, the investigation of choice to discern atherothrombotic from other coronary disease is the same—an ICA. Operationally, many might favour a colocation of subtypes of myocardial infarction that streamlines (i) the investigative path and (ii) subsequent management which is inherently dependent on (i).

## 13. Clinical scenario

**Figure 4:**
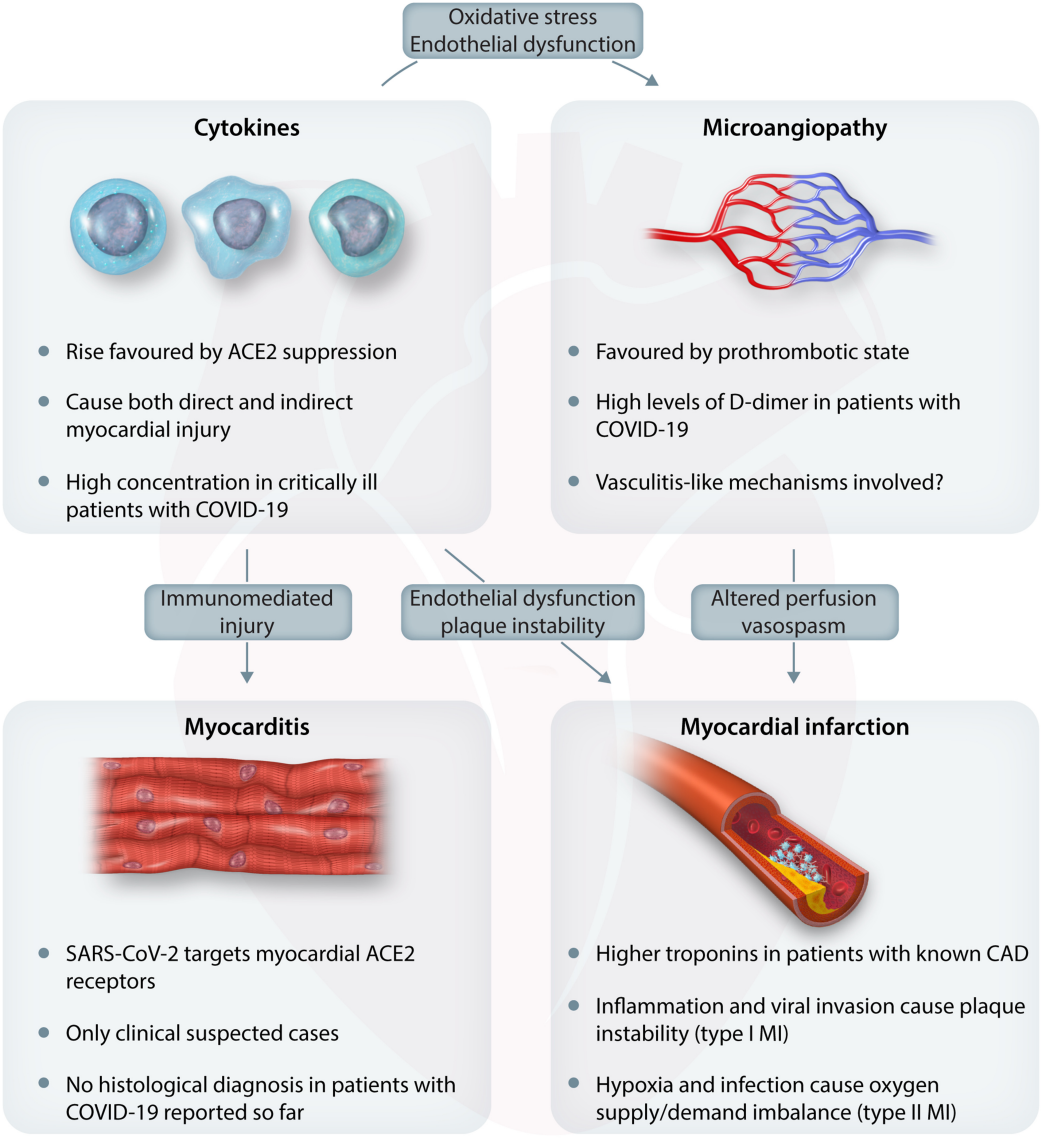
From Tersalvi *et al*.^129^

Imagine the following scenario: a 60-year-old male with a past medical history of hypertension and Type 2 diabetes mellitus is admitted to hospital in April 2020 with breathing difficulty, low oxygen saturation, and relative hypotension at 90/60 mmHg. The patient is noted to be tachycardic and on clinical examination is severely breathless at rest. The chest radiograph is supportive of a diagnosis of COVID-19 pneumonitis, but the differential diagnosis includes pulmonary congestion from acute heart failure, potentially triggered by an acute coronary syndrome. An electrocardiogram obtained at admission is not diagnostic—there are lateral T-wave changes which could be in keeping with left-ventricular hypertrophy or myocardial ischaemia; laboratory parameters demonstrate a low lymphocyte count, high d-dimers and fibrinogen levels, and a high-sensitivity cTnT of 60 ng/L (URL 14 ng/L); renal function demonstrates an acute kidney injury. Symptomatically, the patient describes pain on deep inspiration, but no classic symptoms suggestive of ACS. There is no evidence of ongoing ischaemia through presence of regional wall motion abnormalities on echocardiography. The patient is appropriately treated in a critical care environment and a cardiologist is asked to interpret the elevated cTn level, to guide further management. The patient is pre-disposed to a chronically elevated cTn concentration: systemic hypertension causes an increase in LV afterload. Diabetes mellitus affects micro- and macrovasculature, and the combination of endothelial dysfunction and (previously) stable coronary artery disease in the context of relative hypotension would lead to Type 2 MI. In the context of this patient’s illness, an acute kidney injury could contribute to acute myocardial injury. Even with invasive coronary angiography, the clinician might not be able to rule-out Type 1 MI as a unifying explanation for the elevated cTn concentration in a pro-thrombotic state such as during acute COVID-19. *Figure 4* highlights the various mechanisms contributing to this patient’s illness.

Irrespective of logistics, subjecting a critically ill individual to an invasive procedure does not come without its own risks. Expecting the treating cardiologist to ascribe an elevated biomarker level to a single unifying diagnosis does not only appear to be unduly challenging, given our knowledge of the multi-facetted pathophysiological contributors to acute and chronic troponin elevations but also operationally untenable. The imagined pathophysiological basis of the 4th UDMI often cannot be made to fit clinical reality.

## 14. Does it matter?

The case presented above highlights the uncertainties in everyday acute cardiovascular care—whilst the concepts of the 4th UDMI are pathophysiologically sound, they are challenging to define operationally. What is needed are evolutions in clinical practice that will enable patients to be placed within the pathological framework described in the 4th UDMI. This is necessary for treatments to target the relevant and dominant causative pathology, or pathologies, contributing to the cTn elevation. The future is bright and clinical landscape is changing. Powerful diagnostic techniques such as CTCA and CMR are increasingly available. What is clear is that whatever the cause of elevation cTn indicates prognosis, we just have to learn how to better answer its siren call!

**Conflict of interest:** T.E.K. and B.A. have no conflict of interest to declare. M.M. has interest to declare. M.M. is named as an inventor on a patent held by King’s College London for the detection of cardiac myosin-binding protein C as a biomarker of myocardial injury.

## Funding

This work was supported by grants from the Medical Research Council (London, UK) (G1000737), Guy’s and St Thomas’ Charity (London, UK; R060701, R100404), British Heart Foundation (Birmingham, London; TG/15/1/31518, FS/15/13/31320), and the UK Department of Health through the National Institute for Health Research Biomedical Research Centre award to Guy’s & St Thomas’ National Health Service Foundation Trust. Dr Kaier is funded through an National Institute for Health Research clinical lectureship (CL-2019-17-006).
